# Biological characteristics of manganese transporter MntP in *Klebsiella pneumoniae*

**DOI:** 10.1128/msphere.00377-24

**Published:** 2024-06-18

**Authors:** Wei Peng, Yafei Xu, Yilin Yin, Jichen Xie, Renhui Ma, Guoyuan Song, Zhiqiang Zhang, Qiuhang Quan, Qinggen Jiang, Moran Li, Bei Li

**Affiliations:** 1 School of Basic Medicine, Hubei University of Medicine, Shiyan, Hubei, China; 2 Biomedical Research Institute, Hubei University of Medicine, Shiyan, Hubei, China; The University of Arizona, Tucson, Arizona, USA

**Keywords:** *Klebsiella pneumoniae*, efflux pump, MntP, oxidative stress, metal homeostasis

## Abstract

*Klebsiella pneumoniae* is an important opportunistic pathogen that causes a variety of infections. It is critical for bacteria to maintain metal homeostasis during infection. By using an isogenic *mntP* deletion mutant of *K. pneumoniae* strain NTUH-K2044, we found that MntP was a manganese efflux pump. Manganese increased the tolerance to oxidative stress, and oxidative stress could increase the intracellular manganese concentration. In oxidative stress, the *mntP* deletion mutant exhibited significantly higher sensitivity to manganese. Furthermore, iron could increase the tolerance of the *mntP* deletion mutant to manganese. Inductively coupled plasma mass spectrometry analysis revealed that the *mntP* deletion mutant had higher intracellular manganese and iron concentrations than wild-type and complementary strains. These findings suggested that iron could increase manganese tolerance in *K. pneumoniae*. This work elucidated the role of MntP in manganese detoxification and Mn/Fe homeostasis in *K. pneumoniae*.

**IMPORTANCE:**

Metal homeostasis plays an important role during the process of bacterial infection. Herein, we revealed that MntP was involved in intracellular manganese homeostasis. Manganese promoted resistance to oxidative stress in *Klebsiella pneumoniae*. Furthermore, we demonstrated that the *mntP* deletion mutant exhibited significantly lower survival under manganese and H_2_O_2_ conditions. Oxidative stress increased the intracellular manganese content of the *mntP* deletion mutant. MntP played a critical role in maintaining intracellular manganese and iron concentrations. MntP contributed to manganese detoxification and Mn/Fe homeostasis in *K. pneumoniae*.

## INTRODUCTION

*Klebsiella pneumoniae* is a Gram-negative bacterium, which thrives ubiquitously in environments, including water, vegetation, insects, and animals (1). *K. pneumoniae* is also an opportunistic pathogen that can cause urinary tract infections, pneumonia, bacteremia, and purulent liver abscesses (2). Numerous factors have been reported to contribute to *K. pneumoniae* virulence, such as siderophores, fimbriae, capsules, and lipopolysaccharides (3). The two main pathotypes of *K. pneumoniae* are classical *K. pneumoniae* and hypervirulent *K. pneumoniae* (hvKp) (4). The hypervirulence phenotype of hvKp is associated with the ~200 kb virulence plasmid which contains genes involved in siderophore and capsule production (5, 6). Metal ions can promote the formation of capsules (7, 8).

Mn is an essential nutrient for nearly all organisms and plays several critical roles in cellular physiology as an enzymatic cofactor (9). Therefore, maintaining intracellular Mn levels is paramount for pathogen survival and replication during infection. Mn has been reported to contribute to the pathogenesis of many bacterial species (10). Mn functions as a cofactor for diverse enzymes involved in central carbon metabolism, translation, signaling, and nucleotide metabolism (11). The most well-known role of Mn is its association with oxidative stress defense (10). During infection, neutrophils and macrophages scavenge divalent metal ions by calprotectin to reduce their contents in the host environment (12). To overcome Mn restriction, bacteria use manganese-specific importers to increase the intracellular Mn concentration (11). However, excessive intracellular Mn levels are harmful to bacteria, so it is important for bacteria to be able to transport Mn to the extracellular environment to maintain intracellular Mn homeostasis (10).

Mn homeostasis is associated with several other metals, especially iron ions. Iron is an essential nutrient for pathogenic microbes; nearly all bacterial species need iron during infection (13). Bacteria have evolved a plethora of mechanisms to get iron from environmental and host stocks to maintain intracellular iron homeostasis (14). In bacteria, it has been reported that Mn influenced iron homeostasis (15). The Mn/Fe ratio is critical for bacteria to resist certain types of stress (16, 17). To date, three types of Mn efflux systems have been reported in prokaryotes. The prokaryotic cation diffusion facilitator Mn exporter MntE was first identified in *Streptococcus pneumoniae* and has been identified in *Staphylococcus aureus*, *Enterococcus faecalis*, and *Streptococcus mutans* (18–21). In *Mycobacterium tuberculosis*, P_1B_-ATPase was first established for Mn export (22). MntP has been characterized for Mn export which lacks homology to other Mn exporters (23, 24). However, little is known about the Mn efflux mechanism in *K. pneumoniae*. Mn homeostasis mechanisms have not been characterized.

In the present study, we focused on the biological characteristics of MntP in *K. pneumoniae* and identified its role in Mn efflux. MntP increased resistance to high concentrations of Mn in *K. pneumoniae*. Furthermore, we revealed the role of MntP in intracellular iron homeostasis. Our findings demonstrated that MntP played a crucial role in Mn and iron homeostasis in *K. pneumoniae*.

## RESULTS

### Bioinformatics analysis of the *mntP* gene

**Fig 1:**
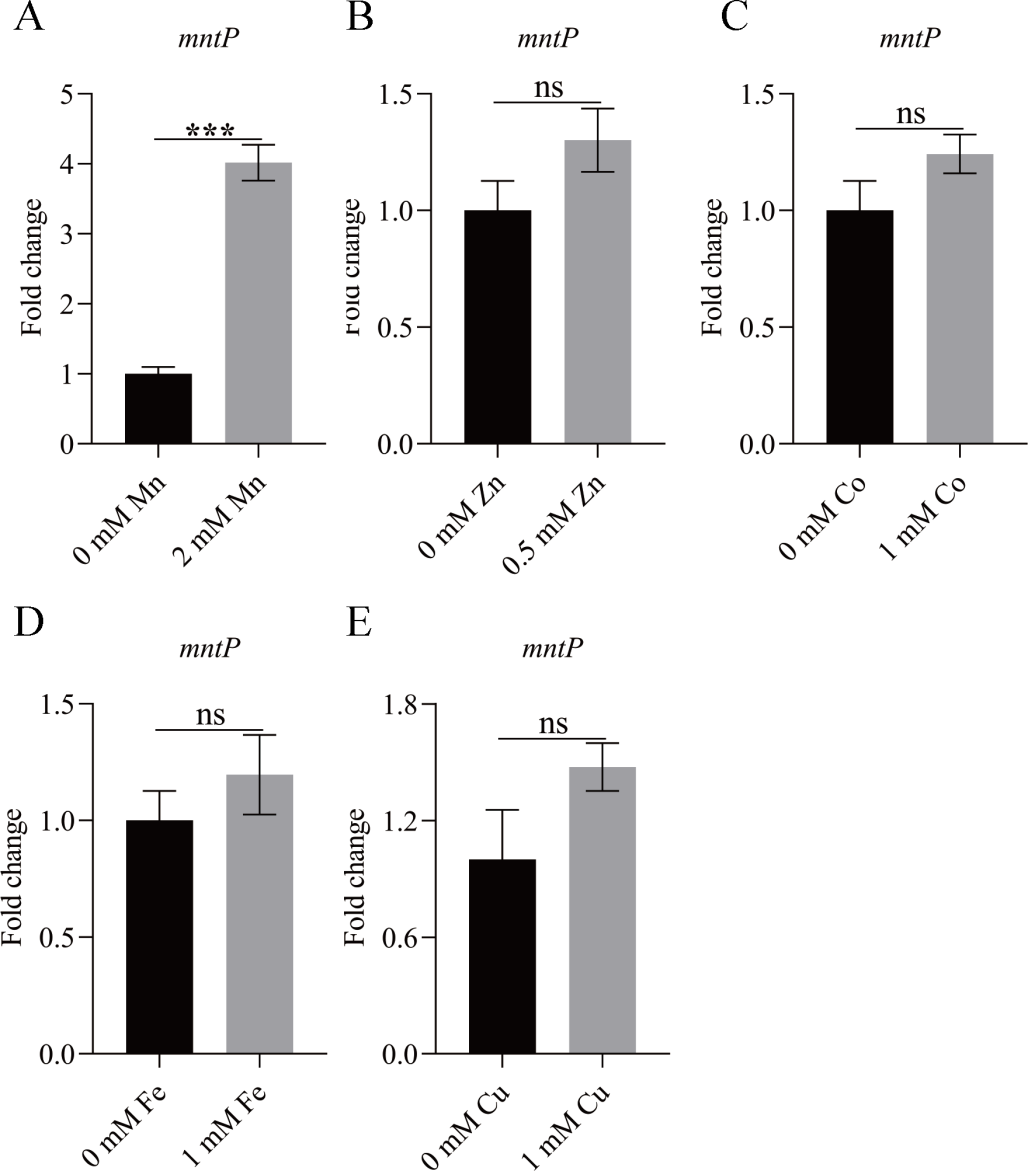
The transcription levels of *mntP* in the wild-type strain in the presence of different metal ions (A) Mn, (B) Zn, (C) Co, (D) Fe, or (E) Cu. The gene expression levels were calculated using the 2^−ΔΔCt^ method with 16S rRNA as the reference gene. The data are expressed as mean ± standard deviation of three independent experiments. Statistically significant differences were determined via Student’s *t*-test (^***^P < 0.001). ns, not significant.

We aimed to identify potential candidate genes involved in the detoxification of excess Mn in *K. pneumoniae*. BlastP analysis revealed that the *KP1_RS16140* gene in *K. pneumoniae* shared 81.91%, 81.38%, and 57.75% amino acid sequence identity with MntP from *Escherichia coli*, *Salmonella enterica*, and *Pseudomonas aeruginosa*, respectively (Fig. S1) (25, 26). The *KP1_RS16140* gene was named *mntP* in *K. pneumoniae*. In order to study the function of *mntP*, the transcription level of *mntP* was measured. As shown in Fig. 1, the transcription level of *mntP* was increased upon exposure to 2 mM Mn, not other metal ions. Furthermore, we constructed the *mntP* deletion mutant and complementary strains (Fig. S2). These data demonstrated that MntP might contribute to Mn detoxification in *K. pneumoniae*.

### *K. pneumoniae* requires *mntP* for Mn detoxification

**Fig 2:**
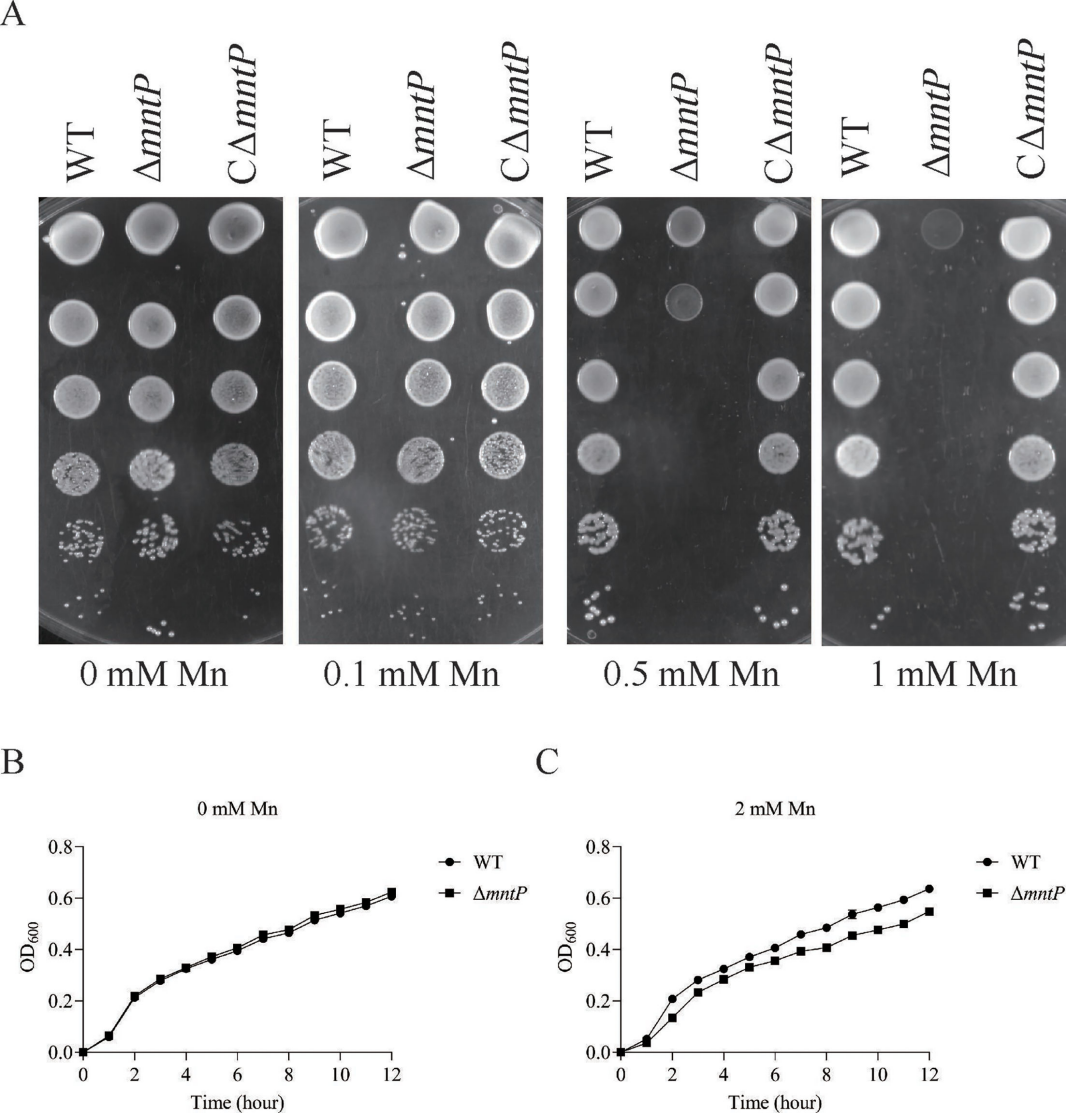
MntP is involved in *K. pneumoniae’s* resistance to manganese toxicity. (**A**) Spot dilution assays of WT, Δ*mntP*, and CΔ*mntP* strains with different manganese concentrations (0, 0.1, 0.5, or 1 mM). (**B and C**) Growth curves of WT and Δ*mntP* strains in the presence of 0 or 2 mM Mn.

To verify the role of *mntP* for Mn detoxification, we test the tolerance of Δ*mntP* to exogenous Mn. As shown in Fig. 2A, the inactivation of *mntP* in *K. pneumoniae* resulted in substantially decreased growth on solid medium in the presence of 0.5 or 1 mM Mn. The Δ*mntP* had similar growth properties to the WT strain supplemented with 0 mM Mn, but Δ*mntP* exhibited a slower growth than the WT strain in medium supplemented with 2 mM Mn (Fig. 2B and C).

**Fig 3:**
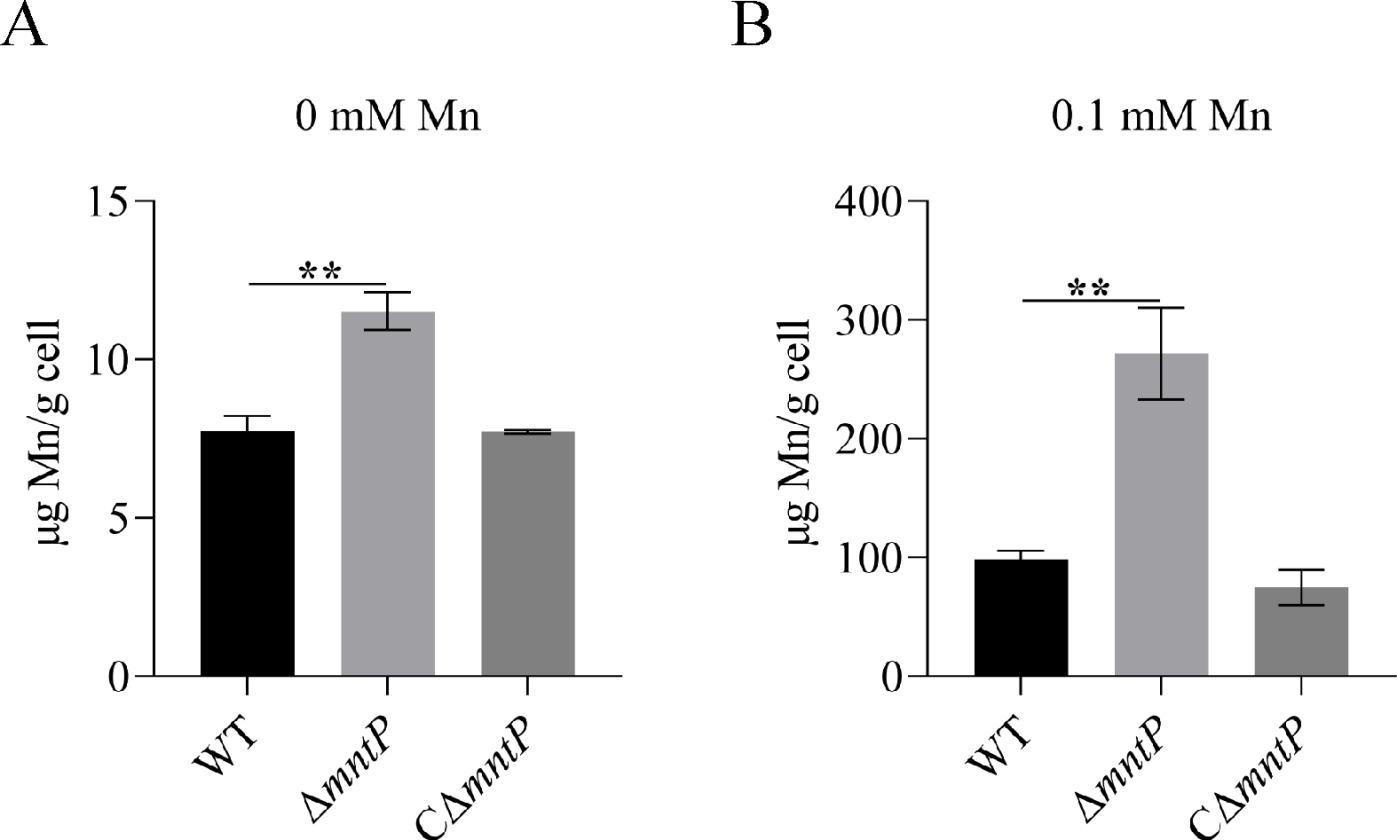
(A and B) Intracellular manganese content of WT, Δ*mntP*, and CΔ*mntP* strains in the presence of 0 or 0.1 mM Mn. The data are expressed as mean ± standard deviation of three independent experiments. Statistically significant differences were determined via Student’s *t*-test (^**^P < 0.01).

Furthermore, to verify the primary function of *mntP* in the process of Mn transport, we conducted inductively coupled plasma mass spectrometry analysis to quantify the intracellular Mn content in WT, Δ*mntP*, and CΔ*mntP* (complementary strain of Δ*mntP*) strains. As shown in Fig. 3A and B, Δ*mntP* accumulated more Mn than WT and CΔ*mntP*. These data suggested that MntP contributed to Mn detoxification and Mn export.

### Oxidative stress promotes Mn accumulation

**Fig 4:**
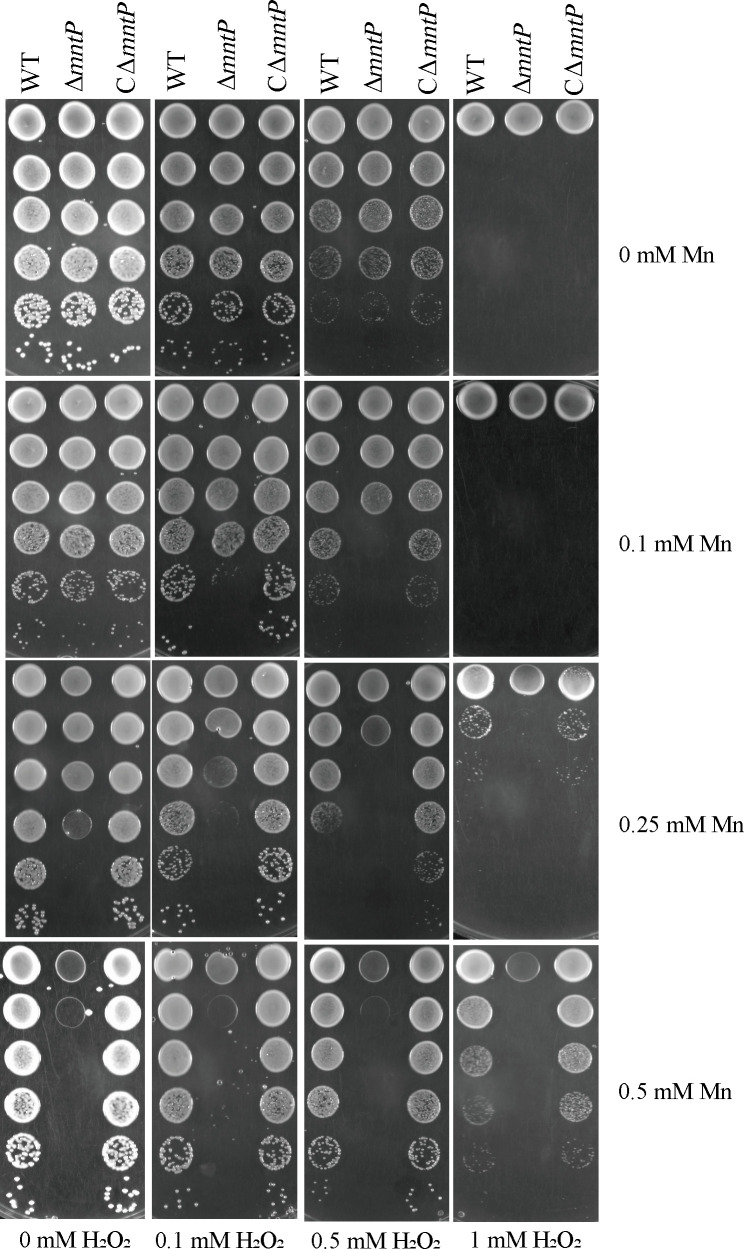
Growth under oxidative stress in combination with manganese. Spot dilution assays of WT, Δ*mntP*, and CΔ*mntP* strains in the presence of different concentrations of manganese (0, 0.1, 0.25, or 0.5 mM; vertical axis) and H_2_O_2_ (0, 0.1, 0.5, or 1 mM; horizontal axis).

**Fig 5:**
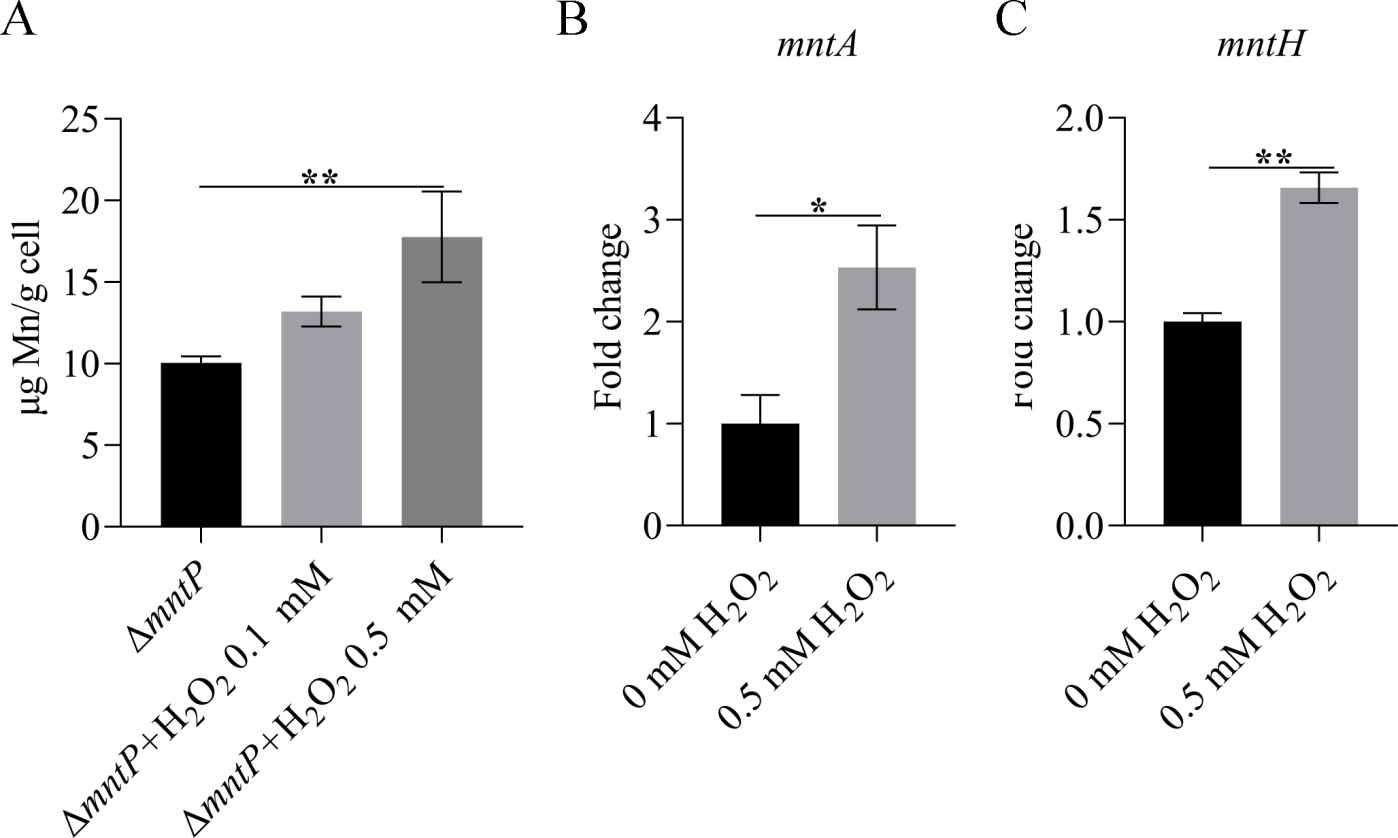
(**A**) Intracellular manganese content of strain Δ*mntP* in the presence of 0, 0.1, or 0.5 mM H_2_O_2_. (B and C) The transcription levels of Mn importers (*mntA* and *mntH*) in the wild-type strain in the presence of 0.5 mM H_2_O_2_. The gene expression levels were calculated using the 2^−ΔΔCt^ method with 16S rRNA as the reference gene. The data are expressed as the mean ± standard deviation of three independent experiments. Statistically significant differences were determined via Student’s *t*-test (^*^P < 0.05; ^**^P < 0.01).

A previous study has indicated that Mn played an important role in resistance to oxidative stress (27). Oxidative stress can increase the uptake of Mn by bacteria. The intracellular reactive oxygen species (ROS) level of *K. pneumoniae* was increased when the medium was supplemented with H_2_O_2_ (Fig. S3). Here, WT, Δ*mntP*, and CΔ*mntP* strains were cultured on a solid medium supplemented with Mn or H_2_O_2_. As shown in Fig. 4, Mn could increase the tolerance of *K. pneumoniae* to H_2_O_2_. 0.1 mM H_2_O_2_ could restrict Δ*mntP* growth at a Mn concentration of 0.1 mM, which had no influence on Δ*mntP* growth in the absence of H_2_O_2_. Furthermore, the intracellular Mn contents were increased upon H_2_O_2_ exposure (Fig. 5A). The transcriptional levels of Mn importers (*mntA* and *mntH*) were increased under H_2_O_2_ (Fig. 5B and C). These data revealed that Mn increased oxidative stress tolerance and that the intracellular Mn concentration was increased under oxidative stress in *K. pneumoniae*.

### Accumulation of intracellular Mn increases the intracellular iron level

**Fig 6:**
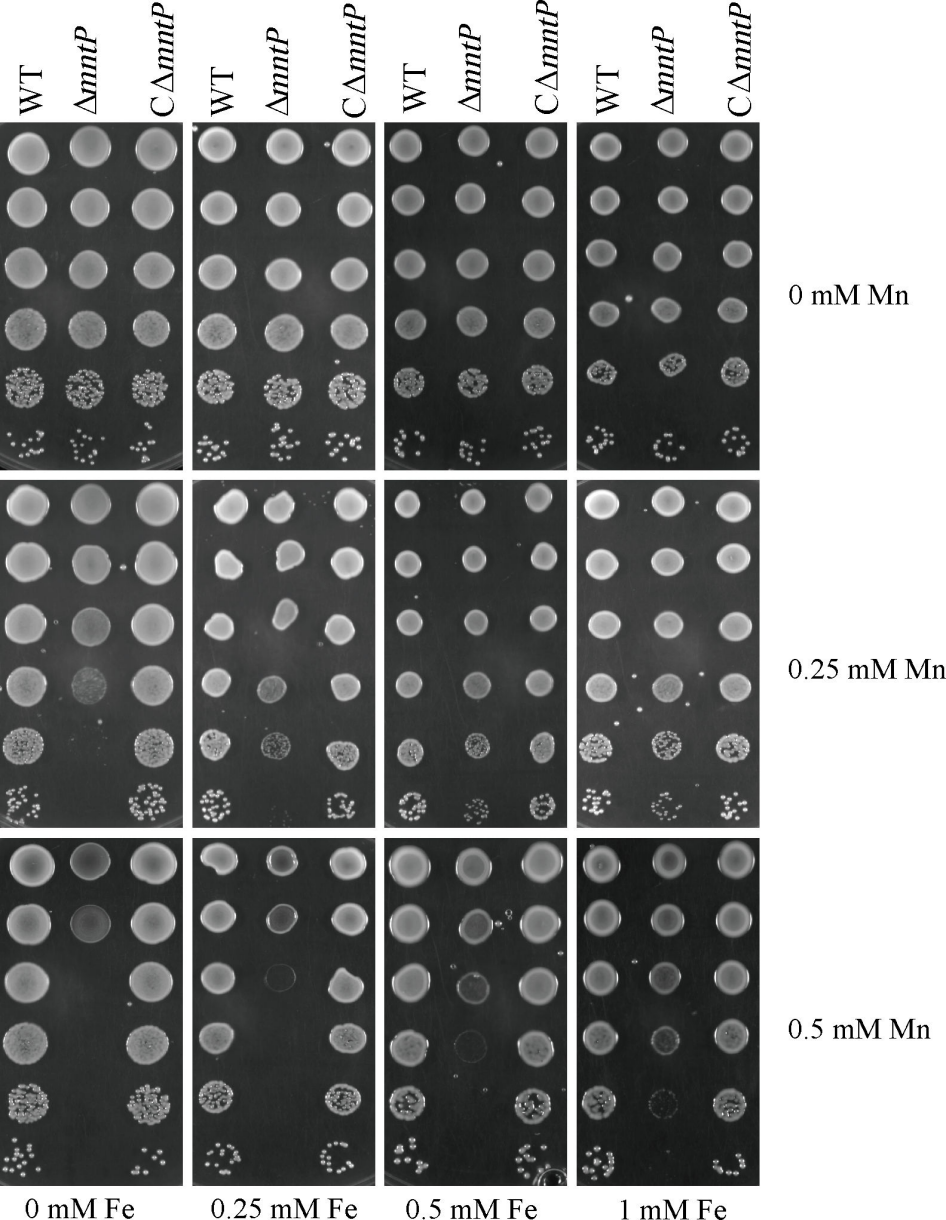
Growth under manganese stress in combination with iron. Spot dilution assays of WT, Δ*mntP*, and CΔ*mntP* strains in the presence of different concentrations of manganese (0, 0.25, or 0.5 mM; vertical axis) and iron (0, 0.25, 0.5, or 1 mM; horizontal axis).

**Fig 7:**
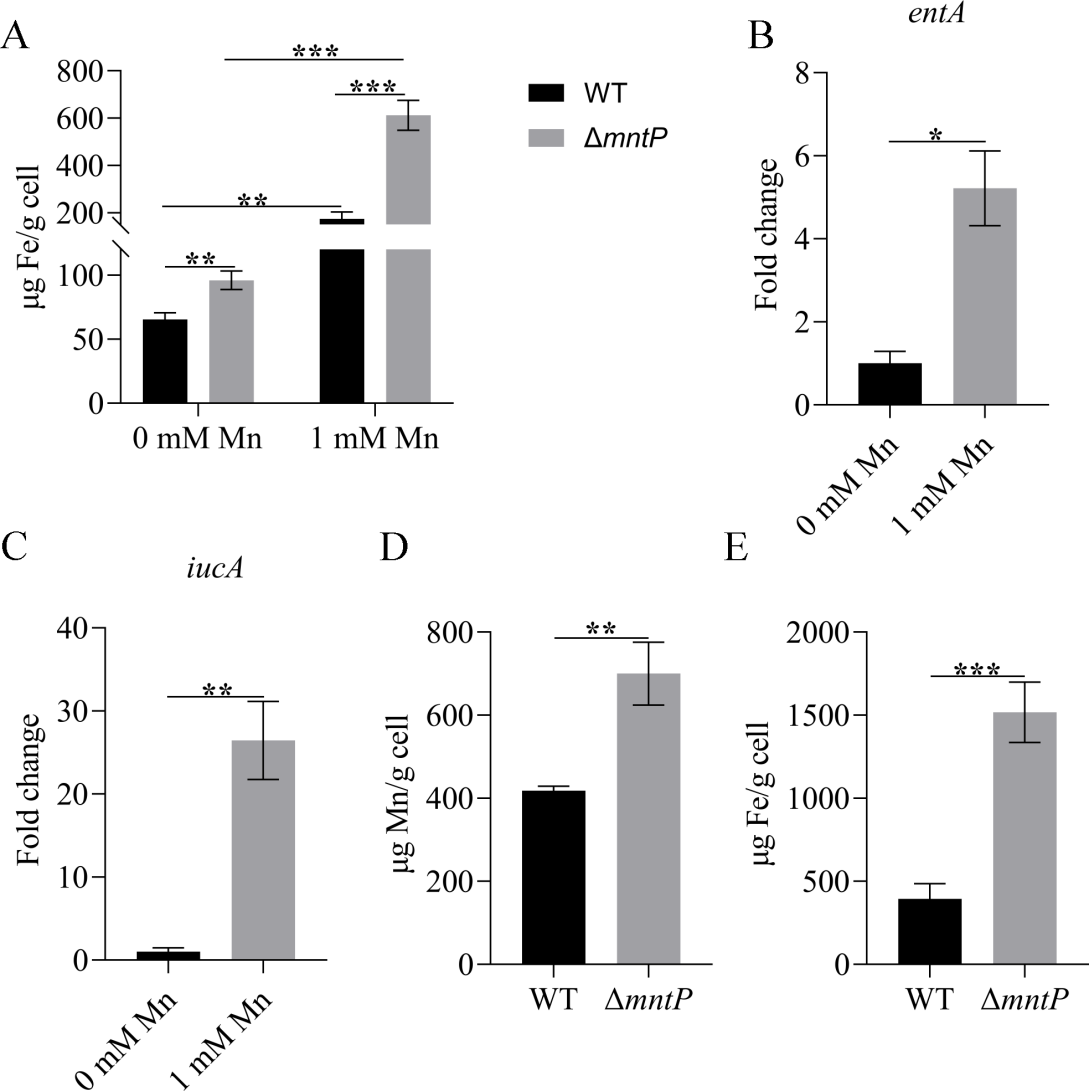
(**A**) Intracellular iron content of WT, Δ*mntP*, and CΔ*mntP* in the presence of 0 or 0.1 mM manganese. (B and C) The transcription levels of Fe importers (*entA* and *iucA*) in the wild-type strain in the presence of 1 mM Mn. The gene expression levels were calculated using the 2^−ΔΔCt^ method with 16S rRNA as the reference gene. (D and E) Intracellular Mn or Fe contents in the presence of 1 mM Mn and 1 mM Fe. The data are expressed as the mean ± standard deviation of three independent experiments. Statistically significant differences were determined via Student’s *t*-test (^*^P < 0.05; ^**^P < 0.01; ^***^P < 0.001).

Bacteria need to maintain ion homeostasis, and intracellular metal ion homeostasis is crucial for the pathogenicity of bacteria (28). A previous study has demonstrated that bacteria regulated the Mn/Fe ratio through Mn transport (24). To determine whether iron could increase Mn tolerance in *K. pneumoniae*, we determined the tolerance to Mn in the presence of iron. As shown in Fig. 6, iron could increase the tolerance of *K. pneumoniae* to Mn. We hypothesized that inactivation of *mntP* would affect the intracellular iron concentration in *K. pneumoniae*. Therefore, we investigated whether Δ*mntP* increased its Mn tolerance by increasing iron uptake. As shown in Fig. 7A, the intracellular iron content in Δ*mntP* was higher than that in the WT strain. Furthermore, the transcriptional levels of iron importers (*entA* and *iucA*) were increased under Mn stress (Fig. 7B and C). The intracellular Mn and iron contents in Δ*mntP* grown with high Mn/high Fe were higher than that of the WT strain (Fig. 7D and E). These data revealed that Mn contributed to Fe homeostasis of *K. pneumoniae*.

## DISCUSSION

As an essential nutrient, Mn is a beneficial metal ion for bacterial pathogens (20). Mn homeostasis is critical to bacterial pathogenicity (29). The goal of this study was to investigate the mechanism of Mn homeostasis in *K. pneumoniae*. During infection, it is important for bacterial pathogens to adapt to the host environment (30). During host-pathogen interactions, the host generates an “oxidative burst” to eliminate pathogenic bacteria (30). The ability to resist oxidative stress is extremely important for bacteria (31). However, an overload of Mn ions can also cause toxicity in bacteria, so bacteria use Mn efflux pumps to control intracellular Mn concentrations (26). Here, we identified a Mn efflux pump, MntP, which contributed to Mn homeostasis in *K. pneumoniae*.

Mn efflux by MntP has been studied in some bacteria (23, 24, 26), but there is no report about Mn efflux in *K. pneumoniae*. In the present study, we revealed that MntP was required for Mn detoxification in *K. pneumoniae*. Upon exposure to 2 mM Mn, the transcription level of the Mn pump gene *mntP* was increased, in accordance with previous studies (21, 32).

Mn can increase the antioxidant capacity of bacteria by a variety of mechanisms (10). Bacteria can increase the intracellular Mn concentration under oxidative stress conditions (27). Mn can increase the ability of bacteria to resist oxidative stress (27, 33). Herein, the results showed that Mn could increase the ability of *K. pneumoniae* to resist oxidative stress (Fig. 4). Oxidative stress can promote intracellular Mn accumulation (34). Furthermore, oxidative stress could increase the intracellular Mn content of *K. pneumoniae* (Fig. 5). However, Δ*mntP* was more sensitive to H_2_O_2_ in the presence of Mn. The previous study has reported that excess manganese leads to constitutive repression of PerR-regulated peroxide defenses (33). When manganese export of bacteria is disrupted, the strain is not able to defend against exogenously produced hydrogen peroxide in excess manganese (33).

In bacteria, Mn can control iron homeostasis (35). Gut bacteria acquire essential metal ions, like Mn and iron, which are associated with susceptibility to infection (36). *K. pneumoniae* can infect its host through the gut (37). Our findings showed that iron could increase the tolerance of Δ*mntP* to Mn (Fig. 6). In agreement with previous studies, we also observed that the intracellular iron contents were increased in Δ*mntP*. Thus, MntP played a significant role in Mn homeostasis and in maintaining the manganese-to-iron ratio in *K. pneumoniae*. Curiously, the Δ*mntP* mutant showed significantly higher quantities of intracellular Fe. However, some studies have proved that increased intracellular Mn resulted in decreased Fe for *E. coli* and *S. aureus* (20, 25). During aerobic conditions, the concentration of Fe increased when *S. pneumoniae* was Mn stressed (9). Herein, the transcription levels of Fe importers (*entA*, *iucA*) were increased when *K. pneumoniae* was Mn stressed. Mn concentrations can influence the activity of the Fur family transcriptional regulator PerR, which controls the expression of Fe-importer genes (38, 39). It was possible that the increased intracellular Mn bind to Fur, and repressed the expression of Fur. Then, the expressions of Fe importer genes were increased. An adequate intracellular Mn/Fe ratio is critical for bacteria to resist certain types of stress (16, 17).

**Fig 8:**
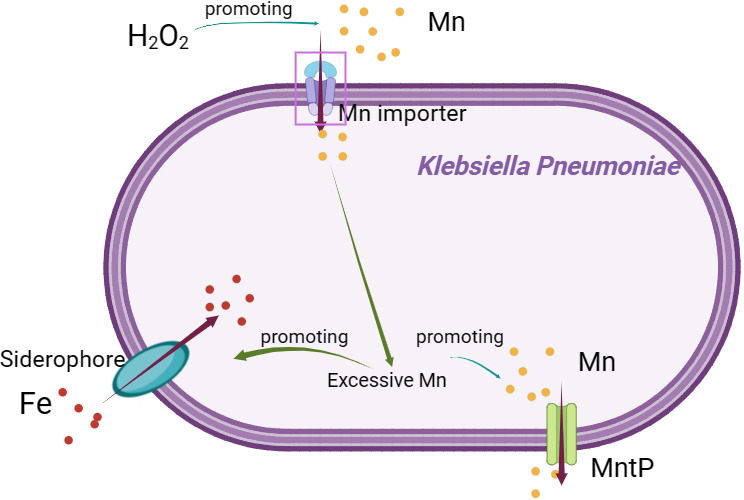
Proposed model for *K. pneumoniae* resistance to Mn toxicity.

In summary, our findings demonstrated that MntP played a crucial role in Mn detoxification. Moreover, Mn could increase the tolerance to oxidative stress in *K. pneumoniae*. MntP contributed to Mn and iron homeostasis (Fig. 8). Taken together, our findings enhanced our understanding of metal ion homeostasis in *K. pneumoniae*.

## MATERIALS AND METHODS

### Bacterial strains, plasmids, primers, and growth conditions

Bacterial strains, plasmids, and primers used in this study are listed in Tables S1 and S2. *K. pneumoniae* was cultured in LB broth or on LB agar plates at 37°C. *E. coli* DH5α competent cells were also grown in LB broth or on LB agar plates at 37°C.

### Bioinformatic analysis

Similarity-based clustering of MntP was carried out using NCBI BLAST. The MntP was from *P. aeruginosa* (QPV52684.1), *E. coli* (NP_416335.4), *Salmonella typhimurium* (UIN61483.1), and *K. pneumoniae* (NC_012731, KP1_RS16140). Multiple alignments were constructed using Clustal Omega (https://www.ebi.ac.uk/Tools/msa/clustalo/). The result was processed using ESPript 3.0 (https://espript.ibcp.fr/ESPript/cgi-bin/ESPript.cgi).

### Construction of the *mntP* deletion mutant and complementary strains

To obtain a markerless deletion mutant, *mntP*-up-F/up-R and *mntP*-down-F/down-R primers were used to separately amplify the upstream and downstream regions of the *mntP* gene by PCR. The upstream and downstream regions were linked by PCR using *mntP*-up-F and *mntP*-down-R primers. The PCR products were cloned into a pKO_3_-Km vector following digestion with corresponding restriction enzymes. Then, the recombinant plasmid was transformed into *K. pneumoniae* strain NTUH-K2044. The mutant strain was obtained as previously described (40). To obtain the complementary strain, the recombinant km-pGEM-T Easy plasmid, which contained the *mntP* gene, was transformed into Δ*mntP*. The *mntP* mutant and complementary strains were confirmed by PCR and reverse transcription PCR.

### RNA extraction and reverse transcription quantitative PCR

WT and Δ*mntP* strains were cultured to mid-log phase supplemented with or without metal ions or H_2_O_2_, and then the strains were collected for RNA extraction. The strain in the growth medium with no supplement served as a control. Total bacterial RNA was extracted using a bacterial total RNA isolation kit (Sangon Biotech, China). Purified RNA was treated with RNase-free DNase I (Qiagen, USA) to eliminate the residual DNA. RNA was reverse transcribed with a SuperScript III first-strand synthesis system (Invitrogen, USA) using random primers in a reaction mixture of 20 µL. AceQ qPCR SYBR green master mix (Bio-Rad, USA) was used to measure mRNA levels according to the manufacturer’s instructions. The relative expression levels were quantified using the comparative threshold cycle (2^−ΔΔCt^) method with 16S rRNA as the endogenous reference.

### Spot dilution assays

WT, Δ*mntP*, and CΔ*mntP* strains were cultured to mid-log phase. Then the cells were harvested by centrifugation and resuspended in phosphate-buffered saline (PBS). The bacterial solutions were serially diluted 10-fold up to a 1:10^6^ dilution, and 5 µL of each dilution was spotted onto LB agar plates supplemented with Mn^2+^, H_2_O_2_, or Fe^3+^. The Mn, H_2_O_2_, or Fe was added to the solid culture medium before it solidified. Then, plates were incubated at 37°C for 12 h.

### Growth curves

Overnight cultures were diluted 1/100 into fresh LB medium with or without Mn at 37°C under shaking conditions, and growth curves were monitored by measuring the optical density at 600 nm by Oy Growth Curves Ab Ltd (Bioscreen C PRO).

### Intracellular manganese content analysis

Intracellular manganese levels were determined as previously described with some modifications (27). The strains were cultured to mid-log phase in LB medium with or without H_2_O_2_, Mn, or Fe. Then the bacteria were harvested by centrifugation, and the cells were washed first three times with PBS containing 0.25 mM EDTA and then three times with PBS. Subsequently, the cells were desiccated at 60°C until dryness. The dry cell weight was measured, and cells were resuspended in 35% (vol/vol) HNO_3_ and boiled at 95°C for 1 h before the removal of debris by centrifugation. Samples were diluted to a final concentration of 1% (vol/vol) HNO_3_ and analyzed by inductively coupled plasma mass spectrometry (ICAP RQ, ThermoFisher).

### Measurement of intracellular ROS

Change in the intracellular ROS was determined using a 2′,7′-dichloro-dihydro-fluorescein diacetate (DCFH-DA) dye (MCE, USA). For our study, *K. pneumoniae* was incubated at 10^8^ CFU/mL and then washed three times with PBS. DCFH-DA (10 µM) was added to *K. pneumoniae* cells for 30 min. Cells were washed three times with PBS. Then, cells were treated with H_2_O_2_ for 1 h. Fluorescence was determined with a microplate reader (SpectraMax i3, Molecular Devices) at an excitation wavelength of 488 nm and an emission wavelength of 525 nm.

### Statistical analysis

GraphPad Prism software was used to analyze the data. The Student’s *t*-test was used to analyze the results. For all tests, a *P*-value of <0.05 was considered to indicate statistical significance.

## SUPPLEMENTAL MATERIAL

Supplemental material msphere.00377-24-s0001.pdf Fig. S1 to S3; Tables S1 and S2.

ASM does not own the copyrights to Supplemental Material that may be linked to, or accessed through, an article. The authors have granted ASM a non-exclusive, world-wide license to publish the Supplemental Material files. Please contact the corresponding author directly for reuse.

## References

[1] Poudel A, Hathcock T, Butaye P, Kang Y, Price S, Macklin K, Walz P, Cattley R, Kalalah A, Adekanmbi F, Wang C. 2019. Multidrug-resistant Escherichia coli, Klebsiella pneumoniae and Staphylococcus spp. in houseflies and blowflies from farms and their environmental settings. Int J Environ Res Public Health 16:3583. doi:10.3390/ijerph16193583 31557837 PMC6801616

[2] Kim SH, Jeon CH, Kim HT, Wi YM. 2023. Clinical characteristics and manifestations in patients with hypermucoviscous Klebsiella pneumoniae bacteremia from extra-hepatobiliary tract infection. Infection 51:689–696. doi:10.1007/s15010-022-01940-6 36271220

[3] Zhu J, Wang T, Chen L, Du H. 2021. Virulence factors in hypervirulent Klebsiella pneumoniae. Front Microbiol 12:642484. doi:10.3389/fmicb.2021.642484 33897652 PMC8060575

[4] Chen J, Zhang H, Liao X. 2023. Hypervirulent Klebsiella pneumoniae. Infect Drug Resist 16:5243–5249. 37589017 10.2147/IDR.S418523 PMC10426436

[5] Shon AS, Bajwa RPS, Russo TA. 2013. Hypervirulent (hypermucoviscous) Klebsiella pneumoniae: a new and dangerous breed. Virulence 4:107–118. doi:10.4161/viru.22718 23302790 PMC3654609

[6] Russo TA, Marr CM. 2019. Hypervirulent Klebsiella pneumoniae. Clin Microbiol Rev 32:e00001-19. doi:10.1128/CMR.00001-19 31092506 PMC6589860

[7] Choby JE, Howard-Anderson J, Weiss DS. 2020. Hypervirulent Klebsiella pneumoniae - clinical and molecular perspectives. J Intern Med 287:283–300. doi:10.1111/joim.13007 31677303 PMC7057273

[8] McFarland AL, Bhattarai N, Joseph M, Winkler ME, Martin JE. 2021. Cellular Mn/Zn ratio influences phosphoglucomutase activity and capsule production in Streptococcus pneumoniae D39. J Bacteriol 203:e0060220. doi:10.1128/JB.00602-20 33875543 PMC8316032

[9] Martin JE, Lisher JP, Winkler ME, Giedroc DP. 2017. Perturbation of manganese metabolism disrupts cell division in Streptococcus pneumoniae. Mol Microbiol 104:334–348. doi:10.1111/mmi.13630 28127804 PMC5380469

[10] Juttukonda LJ, Skaar EP. 2015. Manganese homeostasis and utilization in pathogenic bacteria. Mol Microbiol 97:216–228. doi:10.1111/mmi.13034 25898914 PMC4631260

[11] Bosma EF, Rau MH, van Gijtenbeek LA, Siedler S. 2021. Regulation and distinct physiological roles of manganese in bacteria. FEMS Microbiol Rev 45:fuab028. doi:10.1093/femsre/fuab028 34037759 PMC8632737

[12] Corbin BD, Seeley EH, Raab A, Feldmann J, Miller MR, Torres VJ, Anderson KL, Dattilo BM, Dunman PM, Gerads R, Caprioli RM, Nacken W, Chazin WJ, Skaar EP. 2008. Metal chelation and inhibition of bacterial growth in tissue abscesses. Science 319:962–965. doi:10.1126/science.1152449 18276893

[13] Cassat JE, Skaar EP. 2013. Iron in infection and immunity. Cell Host Microbe 13:509–519. doi:10.1016/j.chom.2013.04.010 23684303 PMC3676888

[14] Kümmerli R. 2023. Iron acquisition strategies in pseudomonads: mechanisms, ecology, and evolution. Biometals 36:777–797. doi:10.1007/s10534-022-00480-8 36508064 PMC10393863

[15] Boyer E, Bergevin I, Malo D, Gros P, Cellier MFM. 2002. Acquisition of Mn(II) in addition to Fe(II) is required for full virulence of Salmonella enterica serovar Typhimurium. Infect Immun 70:6032–6042. doi:10.1128/IAI.70.11.6032-6042.2002 12379679 PMC130432

[16] Daly MJ, Gaidamakova EK, Matrosova VY, Vasilenko A, Zhai M, Venkateswaran A, Hess M, Omelchenko MV, Kostandarithes HM, Makarova KS, Wackett LP, Fredrickson JK, Ghosal D. 2004. Accumulation of Mn(II) in Deinococcus radiodurans facilitates gamma-radiation resistance. Science 306:1025–1028. doi:10.1126/science.1103185 15459345

[17] McEwan AG. 2009. New insights into the protective effect of manganese against oxidative stress. Mol Microbiol 72:812–814. doi:10.1111/j.1365-2958.2009.06700.x 19400770

[18] Rosch JW, Gao G, Ridout G, Wang YD, Tuomanen EI. 2009. Role of the manganese efflux system mntE for signalling and pathogenesis in Streptococcus pneumoniae. Mol Microbiol 72:12–25. doi:10.1111/j.1365-2958.2009.06638.x 19226324 PMC2706702

[19] O’Brien J, Pastora A, Stoner A, Spatafora G. 2020. The S. mutans mntE gene encodes a manganese efflux transporter. Mol Oral Microbiol 35:129–140. doi:10.1111/omi.12286 32129937 PMC8212816

[20] Grunenwald CM, Choby JE, Juttukonda LJ, Beavers WN, Weiss A, Torres VJ, Skaar EP. 2019. Manganese detoxification by MntE is critical for resistance to oxidative stress and virulence of Staphylococcus aureus. mBio 10:e02915-18. doi:10.1128/mBio.02915-18 30808698 PMC6391924

[21] Lam LN, Wong JJ, Chong KKL, Kline KA. 2020. Enterococcus faecalis manganese exporter MntE alleviates manganese toxicity and is required for mouse gastrointestinal colonization. Infect Immun 88:e00058-20. doi:10.1128/IAI.00058-20 32229614 PMC7240088

[22] Padilla-Benavides T, Long JE, Raimunda D, Sassetti CM, Argüello JM. 2013. A novel P(1B)-type Mn2+-transporting ATPase is required for secreted protein metallation in mycobacteria. J Biol Chem 288:11334–11347. doi:10.1074/jbc.M112.448175 23482562 PMC3630897

[23] Waters LS, Sandoval M, Storz G. 2011. The Escherichia coli MntR miniregulon includes genes encoding a small protein and an efflux pump required for manganese homeostasis. J Bacteriol 193:5887–5897. doi:10.1128/JB.05872-11 21908668 PMC3194919

[24] Veyrier FJ, Boneca IG, Cellier MF, Taha MK. 2011. A novel metal transporter mediating manganese export (MntX) regulates the mn to Fe intracellular ratio and Neisseria meningitidis virulence. PLoS Pathog 7:e1002261. doi:10.1371/journal.ppat.1002261 21980287 PMC3182930

[25] Martin JE, Waters LS, Storz G, Imlay JA. 2015. The Escherichia coli small protein MntS and exporter MntP optimize the intracellular concentration of manganese. PLoS Genet 11:e1004977. doi:10.1371/journal.pgen.1004977 25774656 PMC4361602

[26] Ouyang A, Gasner KM, Neville SL, McDevitt CA, Frawley ER. 2022. MntP and YiiP contribute to manganese efflux in Salmonella enterica serovar Typhimurium under conditions of manganese overload and nitrosative stress. Microbiol Spectr 10:e0131621. doi:10.1128/spectrum.01316-21 35019706 PMC8754126

[27] Peng W, Yang X, Wang N, Gao T, Liu Z, Liu W, Zhou D, Yang K, Guo R, Liang W, Chen H, Tian Y, Yuan F, Bei W. 2022. PerR-regulated manganese import contributes to oxidative stress defense in Streptococcus suis. Appl Environ Microbiol 88:e0008622. doi:10.1128/aem.00086-22 35465691 PMC9088288

[28] Jakubovics NS. 2019. An ion for an iron: streptococcal metal homeostasis under oxidative stress. Biochem J 476:699–703. doi:10.1042/BCJ20190017 30819932

[29] Chandrangsu P, Rensing C, Helmann JD. 2017. Metal homeostasis and resistance in bacteria. Nat Rev Microbiol 15:338–350. doi:10.1038/nrmicro.2017.15 28344348 PMC5963929

[30] Christopoulou N, Granneman S. 2022. The role of RNA-binding proteins in mediating adaptive responses in gram-positive bacteria. FEBS J 289:1746–1764. doi:10.1111/febs.15810 33690958

[31] Mortaz E, Alipoor SD, Adcock IM, Mumby S, Koenderman L. 2018. Update on neutrophil function in severe inflammation. Front Immunol 9:2171. doi:10.3389/fimmu.2018.02171 30356867 PMC6190891

[32] Ha N, Lee EJ. 2023. Manganese transporter proteins in Salmonella enterica serovar Typhimurium. J Microbiol 61:289–296. doi:10.1007/s12275-023-00027-7 36862278

[33] Turner AG, Ong C-LY, Gillen CM, Davies MR, West NP, McEwan AG, Walker MJ. 2015. Manganese homeostasis in group A Streptococcus is critical for resistance to oxidative stress and virulence. mBio 6:e00278-15. doi:10.1128/mBio.00278-15 25805729 PMC4453566

[34] Si M, Zhao C, Burkinshaw B, Zhang B, Wei D, Wang Y, Dong TG, Shen X. 2017. Manganese scavenging and oxidative stress response mediated by type VI secretion system in Burkholderia thailandensis. Proc Natl Acad Sci U S A 114:E2233–E2242. doi:10.1073/pnas.1614902114 28242693 PMC5358365

[35] Puri S, Hohle TH, O’Brian MR. 2010. Control of bacterial iron homeostasis by manganese. Proc Natl Acad Sci U S A 107:10691–10695. doi:10.1073/pnas.1002342107 20498065 PMC2890801

[36] Huynh U, Zastrow ML. 2023. Metallobiology of Lactobacillaceae in the gut microbiome. J Inorg Biochem 238:112023. doi:10.1016/j.jinorgbio.2022.112023 36270041 PMC9888405

[37] Osbelt L, Wende M, Almási É, Derksen E, Muthukumarasamy U, Lesker TR, Galvez EJC, Pils MC, Schalk E, Chhatwal P, Färber J, Neumann-Schaal M, Fischer T, Schlüter D, Strowig T. 2021. Klebsiella oxytoca causes colonization resistance against multidrug-resistant K. pneumoniae in the gut via cooperative carbohydrate competition. Cell Host Microbe 29:1663–1679. doi:10.1016/j.chom.2021.09.003 34610293

[38] Guan G, Pinochet-Barros A, Gaballa A, Patel SJ, Argüello JM, Helmann JD. 2015. Pfet, a P1B4 -type ATPase, effluxes ferrous iron and protects Bacillus subtilis against iron intoxication. Mol Microbiol 98:787–803. doi:10.1111/mmi.13158 26261021 PMC4648274

[39] Chiancone E, Ceci P. 2010. The multifaceted capacity of Dps proteins to combat bacterial stress conditions: detoxification of iron and hydrogen peroxide and DNA binding. Biochim Biophys Acta 1800:798–805. doi:10.1016/j.bbagen.2010.01.013 20138126

[40] Ou Q, Fan J, Duan D, Xu L, Wang J, Zhou D, Yang H, Li B. 2017. Involvement of cAMP receptor protein in biofilm formation, fimbria production, capsular polysaccharide biosynthesis and lethality in mouse of Klebsiella pneumoniae serotype K1 causing pyogenic liver abscess. J Med Microbiol 66:1–7. doi:10.1099/jmm.0.000391 27902401

