# Supplementary material for: Biological characteristics of manganese transporter MntP in Klebsiella pneumoniae
Source: mSphere. 2024 Jun 18;9(7):e00377-24. doi: 10.1128/msphere.00377-24 (PMC11288033; doi:10.1128/msphere.00377-24)
Supplement: Supplemental material — Fig. S1 to S3; Tables S1 and S2. [file msphere.00377-24-s0001.pdf]

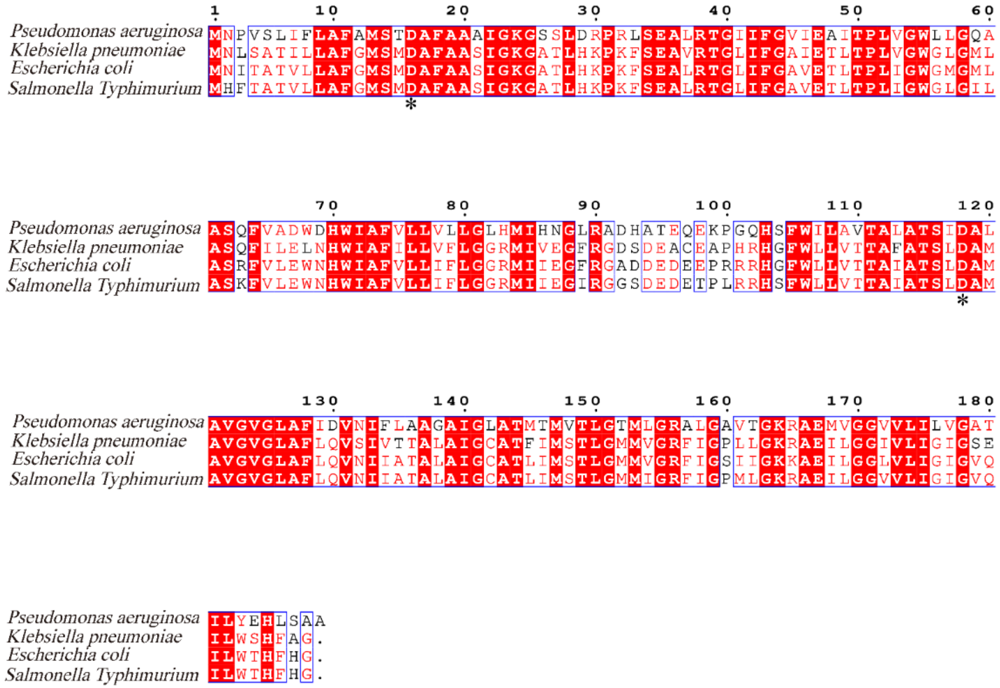

Fig. S1 Multiple sequence alignments of MntP from *K. pneumoniae* with related homologs from *Escherichia coli* (81.91%), *Salmonella enterica* (81.38%), and *Pseudomonas aeruginosa* (57.75%). Identical residues are shown as white letters with a red background; similar residues are shown as red letters with a white background. The black stars (Asp-16 and Asp-118) are the functional important acidic residues for manganese transport.

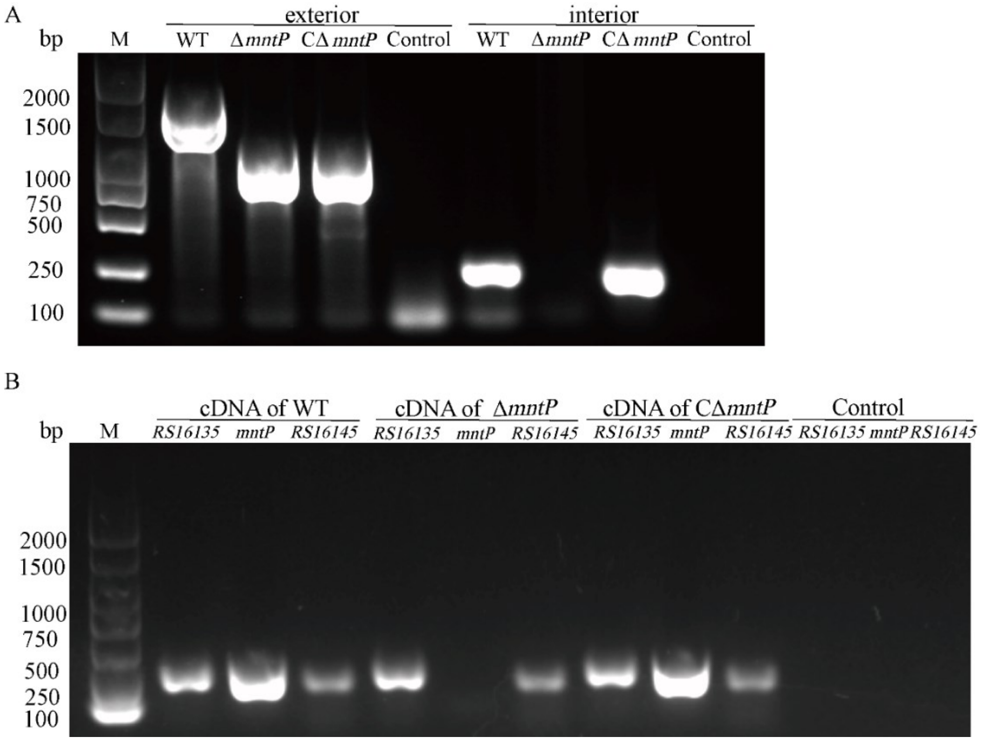

Fig. S2 Identification of *mntP* deletion mutant strain and complementary strain by PCR (A) and RT-PCR (B). (A) The exterior and interior primers of *mntP* gene were used to amplify wild type strain (WT), *mntP* deletion mutant strain (Δ*mntP*), and complementary strain of Δ*mntP* (CΔ*mntP*). M, marker. (B) The interior primers of the upstream of *mntP* (*RS16135*), *mntP*, and the downstream of *mntP* (*RS16145*) were used to detected whether the genes were transcribed normally. The *mntP* of Δ*mntP* could not be transcribed, and the *RS16135* and *RS16145* of Δ*mntP* were transcribed normally. The *RS16135*, *mntP*, and *RS16145* of CΔ*mntP* were transcribed normally.

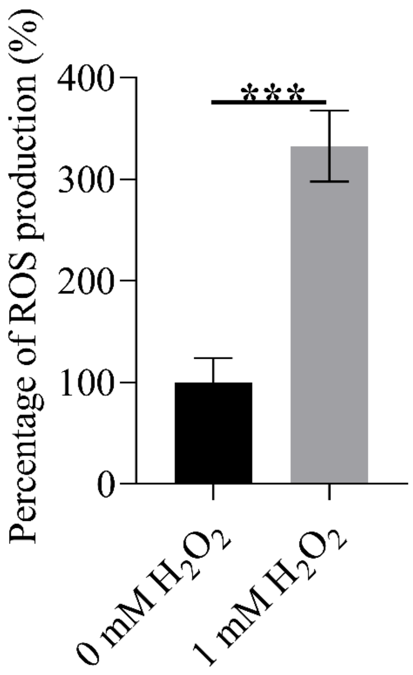

Fig. S3 Reactive oxygen species detection inside *K. pneumoniae.* The data are expressed as the mean ± standard deviation of three independent experiments. Statistically significant differences were determined via Student's *t*-test ($^{***}P < 0.001$).

Supplementary Table 1. Bacterial strains and plasmids used in this work.

Strains or Plasmids	Description[a]	Source or Reference
Strains		
NTUH-K2044	A capsular serotype K1 strain with hypermucoviscosity phenotype, wild-type strain; Ap^r	Our laboratory
Δ*mntP*	Deletion mutant of *mntP* with NTUH-K2044 background	This study
CΔ*mntP*	Complemented strain of *mntP* mutant strain, Kan^r	This study
E. coli DH5α	Cloning host for maintaining the recombinant plasmids	Vazyme
Plasmids		
pKO3	Temperature-sensitive suicide vector, Kan^r	Our laboratory
km-pGEM-T Easy	Vector for *S. suis* complementation, Kan^r	Our laboratory

[a] Kan^r, kanamycin resistant.

Supplementary Table 2. Primers used in this study.

Primer	Sequence (5'-3')	Description
mntP-upstream-F/R	GTATGCGGCCGCAGCCACGGCTATATTTCCCG<br>GCGAAATGGCTCCAGAGGATACATGCCGAAAGCGAGAAGA	Amplification of *mntP* upstream homology arm
mntP-downstream-F/R	TCTTCTCGCTTTCGGCATGTATCCTCTGGAGCCATTTCGC<br>GTATGCGGCCGCGGTGAATTTTTGCGTGGCCT	Amplification of *mntP* downstream homology arm
mntP-interior-F/R	CACCCTGCATAAACCCAAAT<br>CCAGAAGCCAGAAACCATGT	Detection interior of *mntP* mutant strain, amplification of *mntP* used in RT-PCR, and detection the transcription of *mntP* used in qRT-PCR
mntP-complement-F/R	ATGGGCCCATATAGCCAGCGCTATATTA<br>GTATGCGGCCGCTTAACCGGCGAAATGGCTCC	Amplification of *mntP* for constructing the complementary strain
RTPCR-RS16145-F/R	GAAGCTAGCGTCGGAAAATG<br>GGTATTACACCCACGCGTTT	Amplification of *RS16145* used in RT-PCR
RTPCR-RS16135-F/R	TTATTATGCCGAGGCGAAAC<br>GGCGTACGGATCCAGAATAA	Amplification of *RS16135* used in RT-PCR
16SrRNA-F/R	ATGACCAGCCACACTGGAAC<br>CTTCCTCCCCGCTGAAAGTG	An internal region of 16S rRNA
